# Transcriptomic analysis of effects of 1-methylcyclopropene (1-MCP) and ethylene treatment on kiwifruit (*Actinidia chinensis*) ripening

**DOI:** 10.3389/fpls.2022.1084997

**Published:** 2023-01-05

**Authors:** Dasom Choi, Jeong Hee Choi, Kee-Jai Park, Changhyun Kim, Jeong-Ho Lim, Dong-Hwan Kim

**Affiliations:** ^1^ Department of Plant Science and Technology, Chung-Ang University, Anseong, Republic of Korea; ^2^ Food safety and Distribution Research Group, Korea Food Research Institute, Wanju, Republic of Korea; ^3^ Department of Systems Biotechnology, Chung-Ang University, Anseong, Republic of Korea

**Keywords:** ethylene, 1-MCP, kiwifruit, transcriptome, ripening

## Abstract

Ethylene (ET) is a gaseous phytohormone with a crucial role in the ripening of many fruits, including kiwifruit (*Actinidia* spp.). Meanwhile, treatment with 1-methylcyclopropene (1-MCP), an artificial ET inhibitor delays the ripening of kiwifruit. The objective of this study was to determine the effect of ET and 1-MCP application during time-course storage of kiwifruit. In addition, we aimed to elucidate the molecular details underlying ET-mediated ripening process in kiwifruit. For this purpose, we conducted a time-course transcriptomic analysis to determine target genes of the ET-mediated maturation process in kiwifruit during storage. Thousands of genes were identified to be dynamically changed during storage and clustered into 20 groups based on the similarity of their expression patterns. Gene ontology analysis using the list of differentially expressed genes (DEGs) in 1-MCP-treated kiwifruit revealed that the identified DEGs were significantly enriched in the processes of photosynthesis metabolism and cell wall composition throughout the ripening process. Meanwhile, ET treatment rapidly triggered secondary metabolisms related to the ripening process, phenylpropanoid (e.g. lignin) metabolism, and the biosynthesis of amino acids (e.g. Phe, Cys) in kiwifruit. It was demonstrated that ET biosynthesis and signaling genes were oppositely affected by ET and 1-MCP treatment during ripening. Furthermore, we identified a ET transcription factor, *AcEIL* (Acc32482) which is oppositely responsive by ET and 1-MCP treatment during early ripening, potentially one of key signaling factor of ET- or 1-MCP-mediated physiological changes. Therefore, this transcriptomic study unveiled the molecular targets of ET and its antagonist, 1-MCP, in kiwifruit during ripening. Our results provide a useful foundation for understanding the molecular details underlying the ripening process in kiwifruit.

## 1 Introduction

Fruits undergo morphological and physiological changes during growth and maturation. Fruit ripening changes pigment accumulation, texture, softening, and the production of volatile compounds, as well as increases the production of ethylene (ET) by respiration. Fleshy fruits can be divided into two groups depending on their respiration patterns: climacteric and non-climacteric (Leliévre et al., 1997). Kiwifruit (*Actinidia* spp.) is categorized as a climacteric fruit; there is a rapid increase in the production of ET resulting from a ‘climacteric’ burst of respiration (Alexander and Grierson, 2002).

During ripening, fruit undergoes dynamic physiological and biochemical changes. Fruit ripening lead to the accumulation of sugars and changes in fruit texture. For example, fruit softening is accompanied during ripening, which is resulted from the degradation and secondary lignification of cell walls and the degradation of starch during storage (Li et al., 2010; Zhu et al., 2019). During ripening, various phenolic compounds such as anthocyanins and flavonoids are produced, which are derived from the phenylpropanoid pathway during ripening. As a result, fruit color is changed due to the degradation of chlorophyll and accumulation of phenolic compounds like carotenoids, flavonoids, and anthocyanins. Enzymes such as phenylalanine ammonia lyase (PAL), cinnamate 4-hydroxylase (C4H) and 4-coumarate:CoA ligae (4CL) constitutes general phenylpropanoids pathway and then subsequently branched into different biosynthesis pathways for a diversity of end products such as lignin, anthocyanins, flavonols, proanthocyanidins against adverse biotic and abiotic stress conditions (Singh et al., 2010).

These changes are caused by transcriptional reprogramming of genes related to fruit quality during ripening. RNA-sequencing (RNA-seq) technology facilitated a high-throughput transcriptomic analysis on ripening process of flesh fruits including apple (Nawaz et al., 2021), banana (Pathak et al., 2003), strawberry (Tian et al., 2000), and kiwifruit (Asiche et al., 2018; Salazar et al., 2021). These studies showed that many of differentially expressed genes along fruit ripening are involved in the photosynthesis, cysteine and methionine metabolism, phenylpropanoid biosynthesis, amino acid metabolism, starch and sucrose metabolism, and plant hormone signal transduction.

Among plant hormones, ET is the key player that regulates many aspects of fruit ripening (Liu et al., 2015). For example, softening of fruit is caused by modifications in the cell wall, such as reduction in intercellular adhesion, depolymerization of pectins and hemicellulose, and loss of pectic galactose side chains (Brummell and Harpster, 2001). ET induces the expression of several cellular metabolic enzymes, pectin methyl esterase, pectate lyase, polygalacturonase, xyloglucan transglucosylase/hydrolase, and expansin, which are involved in cell wall degradation (Iqbal et al., 2017; Brumos, 2021). For instance, pectin methylesterase (PME) is a hydrolytic enzyme of pectin, one of the most abundant macromolecules in the plant cell wall. Expression of *PME* genes was induced by ET treatment in tomatoes (Wen et al., 2020).

During the last decades, the ET biosynthesis and signaling pathways have been best revealed by intensive studies using the model plant, *Arabidopsis* (Klee, 2004; Kendrick and Chang, 2008). ET biosynthesis is regulated by two major enzymes, known as 1-aminocyclopropane-1-carboxylic acid (ACC) synthase (ACS) and ACC oxidase (ACO). In the ET biosynthesis pathway, S-adenosylmethionine (SAM) is transformed into ACC by the action of ACS, and the ACC is converted to ET *via* ACO (Yang and Hoffman, 1984; Giovannoni, 2004). During the ripening of climacteric fruit, the increased gene expression of ACS and ACO causes a transition to autocatalytic ET production.

In terms of ET signaling, ET receptors perceive the autocatalytic production of ET, and signals are transduced through the cascade to activate several transcription factors and hundreds of related target genes (Adams-Phillips et al., 2004). In *Arabidopsis*, the ET molecule is recognized by five different transmembrane receptors called ETHYLENE RESPONSE/RECEPTOR 1 (ETR1), ETHYLENE RESPONSE/RECEPTOR 2 (ETR2), ETHYLENE RESPONSE SENSOR 1 (ERS1), ETHYLENE RESPONSE SENSOR 2 (ERS2), and ETHYLENE INSENSITIVE 4 (EIN4). In the absence of ET, these ET receptors actively bind with the endoplasmic reticulum-associated Raf-like serine/threonine protein kinase CONSTITUTIVE TRIPLE RESPONSE 1 (CTR1) that constitutively phosphorylates a transmembrane protein named ETHYLENE INSENSITIVE 2 (EIN2). Thus, the ETR/ERS-CTR1 module is a negative regulator of ET signaling (Hua and Meyerowitz, 1998). By contrast, with ET binding to the ETR/ERS-CTR1 receptor module, the CTR1-mediated phosphorylation of EIN2 is not activated (Ju et al., 2012). Unphosphorylated EIN2 undergoes proteolytic cleavage into two fragments (N- and C-terminal fragments) by a yet-unidentified mechanism (Qiao et al., 2012). The N-terminal fragment, which contains multiple transmembrane domains, remains in the endoplasmic reticulum membrane, while the C-terminal fragment is released and translocated to the nucleus and the processing bodies (P-bodies). In the nucleus, the C-terminal EIN2 polypeptide activates transcription factors, such as ETHYLENE INSENSITIVE 3 (EIN3) and EIN3-LIKE 1 (EIL1), which, in turn, trigger expression of downstream ET-responsive genes (Guo and Ecker, 2004). This process leads to many changes to the fruit in addition to starch to sugar accumulation and cell wall degradation leading to softening (Payasi and Sanwal, 2010).

Inhibition of ET biosynthesis is an essential strategy for maintaining the quality of the climacteric fruit and extending the storage period. Application of a non-toxic inhibitor of ET, 1-methylcyclopropene (1-MCP), successfully delayed postharvest ripening of climacteric fruits, including apple (Fan and Mattheis, 1999; Lv et al., 2020), Chinese bayberry (Shi et al., 2018), mango (Reddy et al., 2017), papaya (Shen et al., 2017), and Asian pear (Wang et al., 2021). Similarly, the treatment of 1-MCP to kiwifruit markedly delayed ripening and maintained the fruit quality during storage (Park et al., 2014; Liu et al., 2021). In kiwifruit, ET biosynthetic genes like *AC-SAM1*, *AC-SAM2*, *AC-ACO1*, and *AC-ACO2* genes were shown to be required for the increase of ET production in propylene-treated kiwifruit (Mworia et al., 2010). Also, 1-MCP was shown to inhibit the expression of *KWACO1* and *KWACS1* genes which are related to ET production (Ilina et al., 2010). Furthermore, ET signaling factors in kiwifruit (*Actinidia deliciosa*) such as 4 AdEIN3-like (AdEILs) and 14 ethylene response factors (ERFs) were shown to be dynamically regulated during fruit ripening (Yin et al., 2010). Although the effect of ET and 1-MCP treatments on the physiological and metabolic changes in kiwifruit during ripening were previously examined, the molecular details underlying the postharvest effect of ET and 1-MCP in kiwifruit are still largely understood. Therefore, in this study, we aimed to perform a comparative transcriptomic analysis of kiwifruit before and after postharvest ET or 1-MCP treatment to capture the molecular mechanism involved in ripening and ET biosynthesis under various storage durations.

## 2 Materials and methods

### 2.1 Plant materials and treatment

One of the most popular kiwifruit varieties, the green kiwifruits ‘Hayward’ (dry matter was 15.5~17.5%) were harvested 160 days after full bloom from an orchard in Boseong, Joellanam-do, Korea. Fruit of uniform size and with no physical damage was used for this study. Before ET or 1-MCP treatment, kiwifruits were harvested and named as BT samples. Then, kiwifruit was exposed to 1-MCP (final concentration: 1.4 μL ) or ET (final concentration: 1,000 μL ) at 22°C in sealed plastic containers. After 24 h of treatment, the fruit was transferred to a storage chamber at 22°C. The fruit quality indexes (section 2.2) were measured at 0 days (0D, immediately after transfer to storage chamber), 3 days (3D, stored at storage chamber), 5 days (5D, stored at storage chamber), 7 days (7D, stored at storage chamber), 10 days (10D, stored at storage chamber), 12 days (12D, stored at storage chamber), and 14 days (14D, stored at storage chamber) of storage at storage chamber. For RNA-seq analysis, untreated control, ET-treated, and 1-MCP treated samples harvested at 0D, 3D, 5D, and 7D time points were frozen with liquid nitrogen and used for RNA extraction (section 2.3).

### 2.2 Measurement of fruit firmness, respiration, ET production, and color parameters

Fruit firmness was measured using a texture analyzer (Stable Micro Systems, Godalming, Surrey, UK) equipped with a 5 mm cylindrical probe. The puncture speed and depth were set at 0.5 mm/s and 7 mm, respectively. Puncture measurement was conducted after removing the skin from the central part of the samples, and the highest penetrating value was shown as the fruit firmness. For measuring ET production, fruit samples were located in plastic jars. A headspace sample was taken using a gas-tight syringe for 1 h after sealing the jars and injected into a gas-chromatograph (450-GC, Varian, Middelburg, Netherlands) equipped with a flame ionization detector (FID). CIE L* (lightness), a* (+ redness, − greenness), and b* (+ yellowness, − blueness) values of the fruit surface were recorded with a chromameter (CR-700d, Konica Minolta Optics, Inc., Osaka, Japan).

### 2.3 Total RNA extraction and RNA-Sequencing

RNA-seq analysis was performed on control (untreated), ET- or 1-MCP-treated green kiwifruit ‘Hayward’ stored for 0D, 3D, 5D, and 7D. After an eight-section dissection of the kiwifruit, the middle discs of the fresh fruit were collected, directly frozen in liquid nitrogen, and stored at −80°C in a deep freezer. Frozen middle discs taken from five randomly selected fruit were pooled as a biological replicate. Three biological replicates were prepared for each time point. Total RNAs were extracted using the Spectrum™ Plant Total RNA Kit (Sigma, USA) according to the manufacturer’s instructions. After treatment with DNase I (NEB, USA) to remove contaminated DNA, the quantity of total RNAs was calculated with a Nano-400A spectrophotometer (Allsheng, China). The quality of total RNAs extracted was assessed by electrophoresis on 1% agarose gels as well as with the RNA Nano 6000 Assay Kit in combination with an Agilent Bioanalyzer 2100 (Agilent Technologies, USA). Purified total RNAs were used for RNA-seq library construction using the TruSeq Stranded mRNA LT Sample Prep Kit according to the manufacturer’s instruction (Illumina, USA). Prepared RNA-seq libraries were sequenced on an Illumina NovaSeq 6000 system to generate 101 bp paired-end reads (Macrogen Co., South Korea). In total, 36 RNA-seq libraries were constructed and named as GC-0D-1 (Green kiwifruit, Control, 0 day, replicate 1), GC-0D-2, GC-0D-3, GC-3D-1, GC-3D-2, GC-3D-3, GC-5D-1, GC-5D-2, GC-5D-3, GC-7D-1, GC-7D-2, GC-7D-3, GE-0D-1 (Green kiwifruit, ET-treated, 0 day, replicate 1), GE-0D-2, GE-0D-3, GE-3D-1, GE-3D-2, GE-3D-3, GE-5D-1, GE-5D-2, GE-5D-3, GE-7D-1, GE-7D-2, GE-7D-3, GM-0D-1 (Green kiwifruit, 1-MCP-treated, 0 day, replicate 1), GM-0D-2, GM-0D-3, GM-3D-1, GM-3D-2, GM-3D-3, GM-5D-1, GM-5D-2, GM-5D-3, GM-7D-1, GM-7D-2, and GM-7D-3.

### 2.4 Alignment of RNA-Seq reads

The quality of raw reads was pre-checked using the FastQC software (http://www.bioinformatics.babraham.ac.uk/projects/fastqc). Low-quality reads and adaptor sequences were removed by using the Trimmomatic program (Bolger et al., 2014). Cleaned reads (percentage of reads exhibiting Q30 above 95%) were mapped to the kiwifruit genome (http://kiwifruitgenome.org/node/152174) using TopHat2 (2.0.12) with default parameters (Kim et al., 2013). Mapped reads were then converted to digital counts using featureCounts (Liao et al., 2014).

### 2.5 Identification of differentially expressed genes

The edgeR program was used to extract DEGs (Robinson et al., 2009). DEGs were identified based on an adjusted *P*-value <0.05 and an absolute log_2_|fold change| ≥1. A multi-dimensional scaling (MDS) plot and correlation heatmap data were produced using R packages (ver. 3.6.0). Venn diagram analysis was performed using VENNY (ver. 2.1.0) (https://bioinfogp.cnb.csic.es/tools/venny/). Mapped reads were also converted to bigwig files for visualization using the Integrative Genomics Viewer (IGV) software of the Broad Institute (Thorvaldsdóttir et al., 2013). Gene Ontology (GO) enrichment analysis of DEGs was conducted using ShinyGO (ver. 0.76) (http://bioinformatics.sdstate.edu/go/). Heatmap analysis of DEGs was performed using the MeV program ver. 4.9.0 (Howe et al., 2010).

### 2.6 Quantitative RT-qPCR analysis

Total RNAs (5 μg) were used to synthesize complementary DNAs (cDNAs) using oligo-dT primer and M-MLV reverse transcriptase (Promega, USA). Gene-specific primers were designed by DNA Club software and synthesized by Bionics (South Korea). Primer sequences used in this study are listed in **Supplementary Table S1**. qRT-PCR analysis was performed using the BIOFACT™ 2X Real-Time PCR Master Mix (BioFact, South Korea) on a LineGene 9600 Plus Real-Time PCR system (BioER, China). The PCR reaction conditions were as follows: 12 min denaturation at 95°C, followed by 50 cycles at 95°C for 15 s for denaturation, 60°C for 25 s for annealing, and 72°C for 35 s for extension. *AcACTIN* (Acc08082.1) was used as a reference gene (Atkinson et al., 2009). The 2^−ΔΔ^ *^C^* ^T^ algorithm was employed to quantify the relative transcript level of genes (Livak and Schmittgen, 2001).

### 2.7 Statistical analysis

All statistical analyses in this study were performed using a statistical software package (SAS ver. 9.4; SAS Institute, Inc., Cary, NC, USA). Statistical differences were calculated through one-way analysis of variance (ANOVA) and *post-hoc* Tukey’s test (p < 0.05), and the significance level was assessed at *P* < 0.05. All data results were expressed as mean ± standard deviation of three biological replicates.

## 3 Results

### 3.1 Effect of ET and 1-MCP treatment on fruit quality indexes during storage

**Figure 1:**
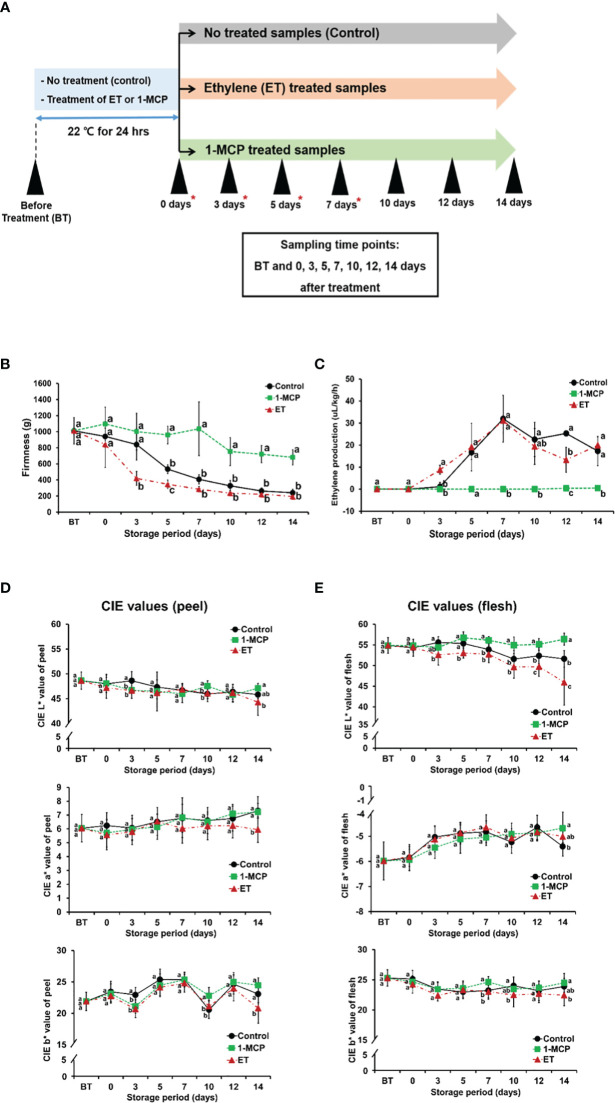
Characterization of firmness, ET amount, color (CIE L*, a*, and b* values) of control, ET-treated, and 1-MCP-treated kiwifruit throughout ripening. **(A)** Schematic diagram showing kiwifruit sampling along ripening time course. Kiwifruit harvested immediately before each treatment was named BT, before treatment. Kiwifruit exposed to ET or 1-MCP, or non-treated (named as control) for 24 h in a chamber, then analyzed without further storage, were named 0D samples. Kiwifruit samples further stored for certain durations (3, 5, 7, 10, 12, and 14 days) were named 3D, 5D, 7D, 10D, 12D, and 14D, respectively. Red asterisks indicate the samples used for RNA-seq analysis. **(B)** Firmness of kiwifruit treated with ET or 1-MCP and non-treated control samples along ripening time course. **(C)** ET production of kiwifruit treated with ET or 1-MCP and non-treated control samples along ripening time course. **(D)** Peel color (L*a*b*) of kiwifruit treated with ET or 1-MCP and non-treated control samples along ripening time course. **(E)** Flesh color (L*a*b*) of kiwifruit treated with ET or 1-MCP and non-treated control samples along ripening time course. **(B–E)** Data were expressed as mean ± standard deviation. One-way analysis of variance (ANOVA) with Tukey’s *post hoc* test was applied to calculate statistical differences. Different lowercase letters indicate significant differences (adjusted *P* < 0.05). ET, ethylene; 1-MCP, 1-methylcyclopropene.

Treatment with ET, a gaseous hormone, is well known to rapidly trigger the ripening of fruits, including kiwifruit, whereas 1-MCP, a non-toxic chemical inhibitor of ET, is demonstrated to prohibit ET-mediated ripening in climacteric fruits (Serek et al., 1995; Liu et al., 2015). To investigate the molecular details underlying ET or 1-MCP effects on kiwifruit ripening by analyzing the genome-wide transcriptional profile, we prepared three different sample groups: control (non-treated), ET-treated, and 1-MCP-treated samples. Each group was analyzed for fruit qualities, including fruit firmness, ET level, and color parameters (L*a*b), on eight different occasions: before treatment (BT), on 0 days (0D; immediately after 24 h-treatment), and post-treatment at 3, 5, 7, 10, 12, and 14 days of storage at 22°C (3D, 5D, 7D, 10D, 12D, and 14D; **Figures 1A–E**). Fruit firmness decreased significantly with storage time (**Figure 1B**). ET treatment accelerated the reduction in fruit firmness, whereas 1-MCP treatment dramatically maintained the fruit firmness throughout the entire time course (**Figure 1B**). Measurement of the endogenous amounts of ET revealed an increase at the 3D time point (8.817 μL/kg/h), but the level became similar to the control samples at later time points (5D, 7D, 10D, and 14D), although temporarily lower at the 12D time point than the control samples (**Figure 1C**). In contrast, the 1-MCP treatment had no significant effect on the ET levels at all tested time points (BT−14D), indicating that 1-MCP can strictly prohibit the biosynthesis of ET in kiwifruit.

Color parameters (L*a*b*) of kiwifruit skin and flesh were measured from harvest (BT) to the end of storage (14D) in all three groups (**Figures 1D, E**). There was no obvious difference in the skin color parameters compared to the control samples at all time points, and the only significant difference in the color of kiwifruit flesh was the L*-value of ET-treated samples, which decreased drastically compared to the control sample. Taken together, these results indicated that the experiments with control and ET- and 1-MCP-treated kiwifruit samples were successfully performed throughout ripening.

### 3.2 Transcriptional changes in kiwifruit during ripening

To dissect the transcriptomic changes in ‘Hayward’ kiwifruit during postharvest ripening, RNA-seq analysis was performed with kiwifruit stored for different durations (0, 3, 5, and 7 days after harvest) and termed GC-0D, GC-3D, GC-5D, and GC-7D, respectively. After filtering of poor-quality reads, paired reads with high-quality Q-values (>30) were aligned to the *Actinidia chinensis* ‘Red5’ reference genome, which was downloaded from the Kiwifruit Genome Database (KGD; https://kiwifruitgenome.org/) (Yue et al., 2020). The MDS plot of green kiwifruit samples across four time points showed group clustering between time point samples, indicating that the RNA-seq libraries were properly constructed and sequenced (**Supplementary Figure S1A**). The correlation heatmap analysis of control samples along the four different time points indicated that the GC-0D and GC-3D samples belonged to one group, and the GC-5D and GC-7D samples belonged to another group, indicating potentially similar transcriptomic profiles between the members of each group (**Supplementary Figure S1B**). This result suggested that dramatic changes in the transcriptome of kiwifruit might occur between 3D and 5D postharvest under our experimental condition.

**Figure 2:**
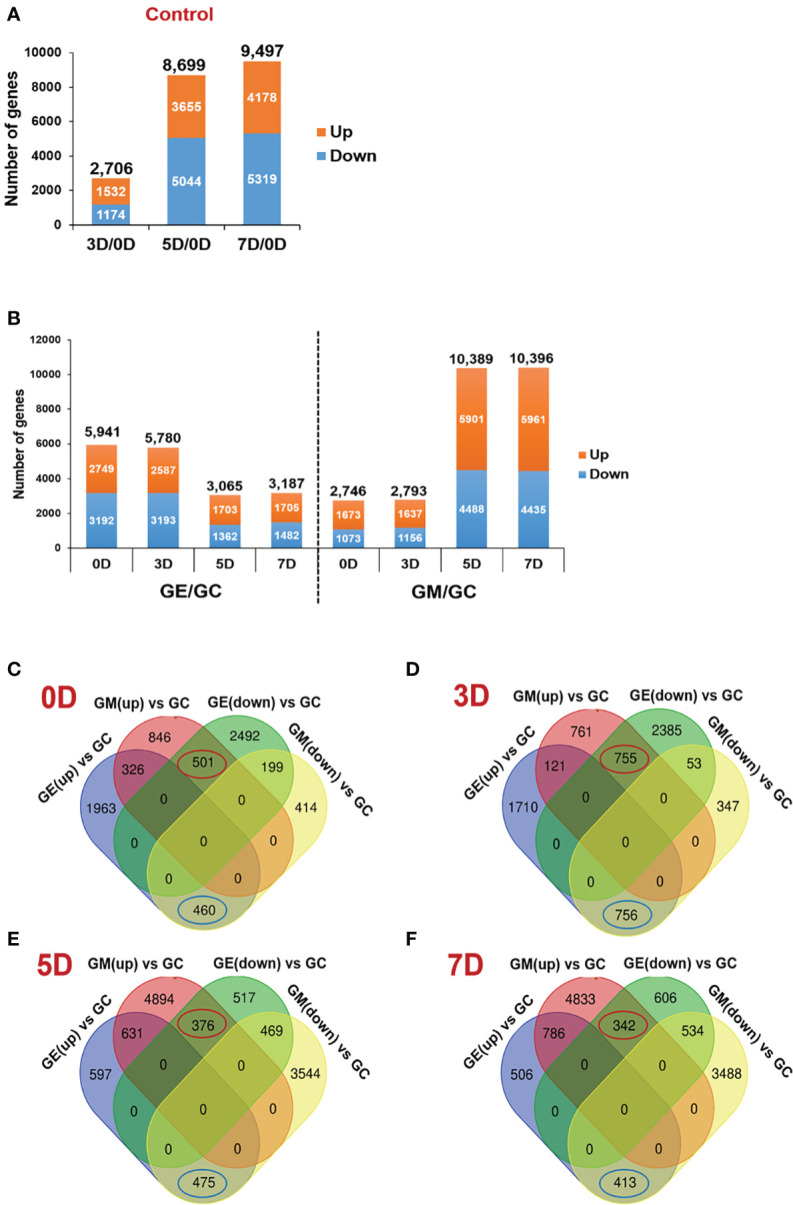
Identification of DEGs between ET-treated and control samples or between 1-MCP-treated samples and control samples. **(A)** Number of DEGs along the ripening time course of kiwifruit at 3, 5, and 7 days (3D, 5D, and 7D), respectively, compared to 0 days (0D) control. **(B)** Number of DEGs along the ripening course of kiwifruit at 0D, 3D, 5D, and 7D between ET-treated (GE) and control samples (GC) or 1-MCP-treated (GM) and control samples, respectively. **(C)** Venn diagram showing the overlapping and uniquely DEGs at 0D, compared between ET-treated sample and 1-MCP-treated sample. A total of 501 genes were upregulated by 1-MCP and downregulated by ET, whereas 460 genes were downregulated by 1-MCP and upregulated by ET. **(D)** Venn diagram showing the overlapping and uniquely DEGs at 3D, compared between ET-treated sample and 1-MCP-treated sample. A total of 755 genes were upregulated by 1-MCP and downregulated by ET, whereas 756 genes were downregulated by 1-MCP and upregulated by ET. **(E)** Venn diagram showing the overlapping and unique DEGs at 5D, compared between ET-treated sample and 1-MCP-treated sample. A total of 376 genes were upregulated by 1-MCP and downregulated by ET, whereas 475 genes were downregulated by 1-MCP and upregulated by ET. **(F)** Venn diagram showing the overlapping and uniquely DEGs at 7D, compared between ET-treated sample and 1-MCP-treated sample. A total of 342 genes were upregulated by 1-MCP and downregulated by ET, whereas 413 genes were downregulated by 1-MCP and upregulated by ET. DEG, differentially expressed gene; ET, ethylene; 1-MCP, 1-methylcyclopropene; GM, green kiwifruit with 1-MCP treatment; GC, green kiwifruit with no treatment (control); GE, green kiwifruit with ET treatment.

A total of 2,706 (1,532 up- and 1,174 downregulated), 8,699 (3,655 up- and 5,044 downregulated) and 9,497 (4,178 up- and 5,319 downregulated DEGs were detected in pairwise comparisons (log_2_|fold change| > 1, *P*-value <0.05) of the 3D, 5D, and 7D samples with the non-stored sample, 0D (**Figure 2A**). In addition, the DEGs were grouped based on the expression profiles during storage, resulting in a total of 20 hierarchical clusters (**Supplementary Figure S2**). Genes belonging to each cluster might be useful as a resource for further investigation to understand the kiwifruit ripening process (**Supplementary Table S2**).

### 3.3 Transcriptomic dynamics of kiwifruit pretreated with ET and 1-MCP during ripening

As a plant hormone, ET plays a crucial role in kiwifruit ripening; thus, we aimed to capture the genome-wide transcriptional effects of ET and 1-MCP treatments on kiwifruit ripening at equivalent time points during storage. Sampling was performed at four different time points (0D, 3D, 5D, and 7D) post-treatment with ET or 1-MCP, and RNA-seq libraries were constructed. It should be emphasized that the 0D samples were those analyzed 24 h after the ET or 1-MCP treatment to examine the short-term effects of ET and 1-MCP. The MDS plot of the control, ET-treated, and 1-MCP-treated samples at each time point showed that samples exposed to the same treatment were closely clustered, indicating that the RNA-seq libraries were well constructed and sequenced (**Supplementary Figure S3**). After mapping to the ‘Red5’ reference genome, DEGs (log_2_|fold change| > 1 and *P*-value < 0.05) were isolated by comparison between control and ET-treated samples or between control and 1-MCP-treated samples (**Figure 2B**, **Supplementary Figure S4**). In ET-treated samples, 5,941, 5,780, 3,065, and 3,187 genes were differentially expressed at 0D, 3D, 5D, and 7D, respectively (**Figure 2B**). This pattern of a higher number of DEGs in the early time points (0D and 3D) than in later time points (5D and 7D) in ET-treated samples was opposite to the pattern displayed by 1-MCP-treated samples, in which the later time point samples (5D and 7D) exhibited substantially higher numbers of DEGs (10,389, and 10,396) compared to the early time point samples (0D and 3D with 2,746 and 2,793 DEGs, respectively) (**Figure 2B**). This contrasting pattern might imply that ET treatment strongly affects kiwifruit during early postharvest storage, whereas 1-MCP affects more sustainably on longer stored samples under our experimental condition.

### 3.4 DEGs upon ET or 1-MCP treatment during ripening

Considering the opposing roles of ET and 1-MCP in fruit ripening, we further analyzed the list of DEGs to extract genes oppositely affected by ET and 1-MCP treatment at individual time points. At 0D, 501 genes were upregulated by 1-MCP treatment and inversely downregulated by ET treatment. Meanwhile, 460 genes were downregulated in 1-MCP-treated samples but upregulated in ET-treated samples (**Figure 2C**). At the later time points of 3D, 5D, and 7D, we identified 755, 376, and 342 genes that were upregulated by 1-MCP and inversely downregulated by ET (**Figures 2D–F**). Meanwhile, 756, 475, and 413 genes were downregulated by 1-MCP and inversely upregulated by ET at 3D, 5D, and 7D, respectively. Information on the DEGs affected by ET and 1-MCP treatment at each time point is listed in **Supplementary Table S3**.

### 3.5 Cell wall genes are significantly affected by ET and 1-MCP treatment

**Figure 3:**
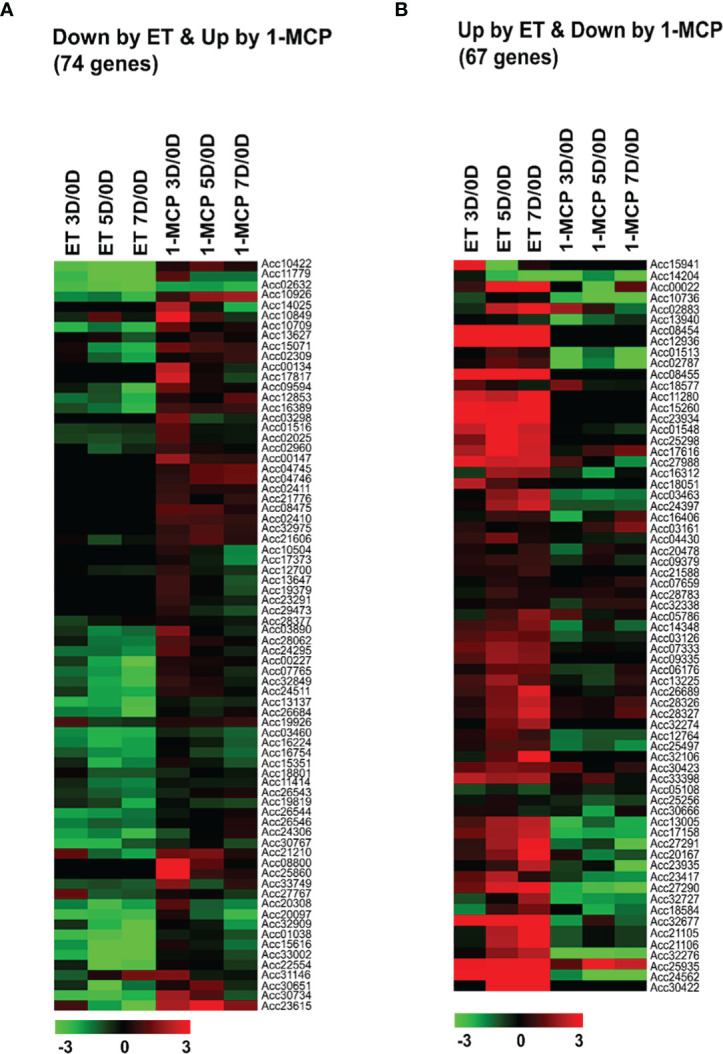
Normalized heatmaps showing expression levels of differentially expressed ‘cell wall’-related genes inversely affected by ET and 1-MCP treatment along ripening time course. **(A)** Seventy-four cell-wall-related genes showed downregulated expression in ET treatment and upregulated expression in 1-MCP treatment. **(B)** Sixty-seven cell-wall-related genes showed upregulated expression in ET treatment and downregulated expression in 1-MCP treatment. Expression level of cell wall-related genes showing differential expression in treated samples stored for 3 vs. 0 days (3D/0D), 5 vs. 0 days (5D/0D), and 7 vs. 0 days (7D/0D). ET, ethylene; 1-MCP, 1-methylcyclopropene.

It is well known that the amount of lignin (an end-product in the phenylpropanoids pathway) is increased during fruit ripening, whereas cellulose/hemicellulose is generally degraded; thus, fruit lignification is accelerated with fruit pigmentation (Zhang et al., 2021). Our results depicting the change in kiwifruit firmness during ripening (**Figure 1B**) are consistent with this pattern. To substantiate these observations, we identified the cell wall genes related to cellulose/hemicellulose degradation as well as cell wall lignification showing differential expression between ET- and 1-MCP-treated samples during the ripening phase. As a result, the expression patterns of a total of 131 genes were detected in ET- and 1-MCP-treated samples from 0D to 7D (**Supplementary Table S4**). Many genes, such as polygalacturonases and expansins, which are related to fruit ripening, were highly expressed in ET-treated samples but lowly expressed in 1-MCP-treated samples throughout the ripening process (**Figures 3A, B**). This result indicates that cell-wall-related genes are one of the targets of ET and 1-MCP during kiwifruit ripening. Consistent with the RNA-seq data, the qRT-PCR analysis on *UDP-glucose pyrophosphorylase 3* (Acc01548), a cell-wall-related gene, showed that the expression level increased in ET-treated samples during storage, whereas it was repressed in 1-MCP-treated samples (**Supplementary Figure S5A**).

### 3.6 GO categories associated with photosynthesis and gene silencing were enriched in 1-MCP-treated samples

**Figure 4:**
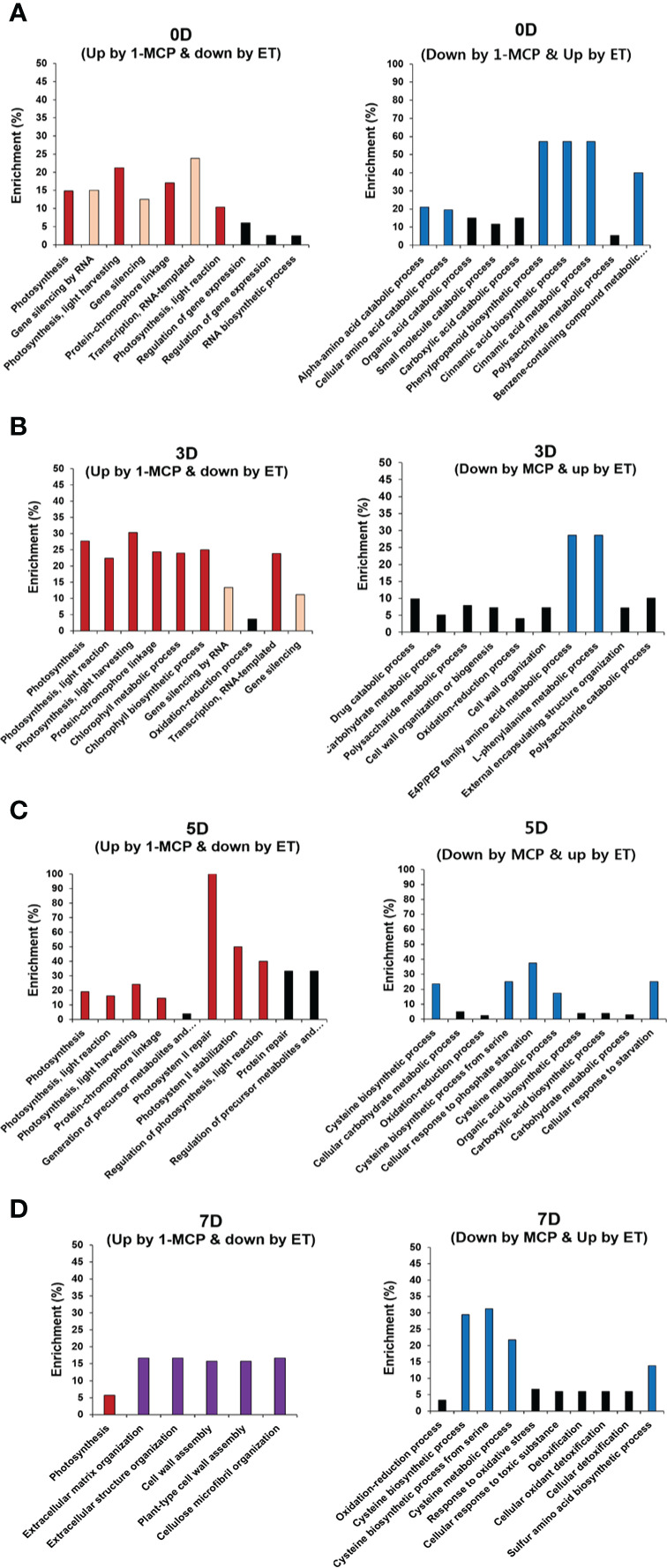
GO enrichment analysis of DEGs oppositely affected by ET and 1-MCP throughout ripening. Top 10 functional GO categories of DEGs showing inverse expression in kiwifruit exposed to ET and 1-MCP treatment and then stored for **(A)** 0 days (0D), **(B)** 3 days (3D), **(C)** 5 days (5D), and **(D)** 7 days (7D). A−D Left panels represent functional GO categories using genes showing upregulated expression by 1-MCP and downregulated expression by ET treatment, right panels represent functional GO categories using genes showing downregulated expression by 1-MCP and upregulated expression by ET. GO categories related to photosynthesis (red bars), gene silencing (yellow bars), the cell wall (purple bars), and cysteine biosynthesis (blue bars). GO, gene ontology; DEG, differentially expressed gene; ET, ethylene; 1-MCP, 1-methylcyclopropene.

**Figure 5:**
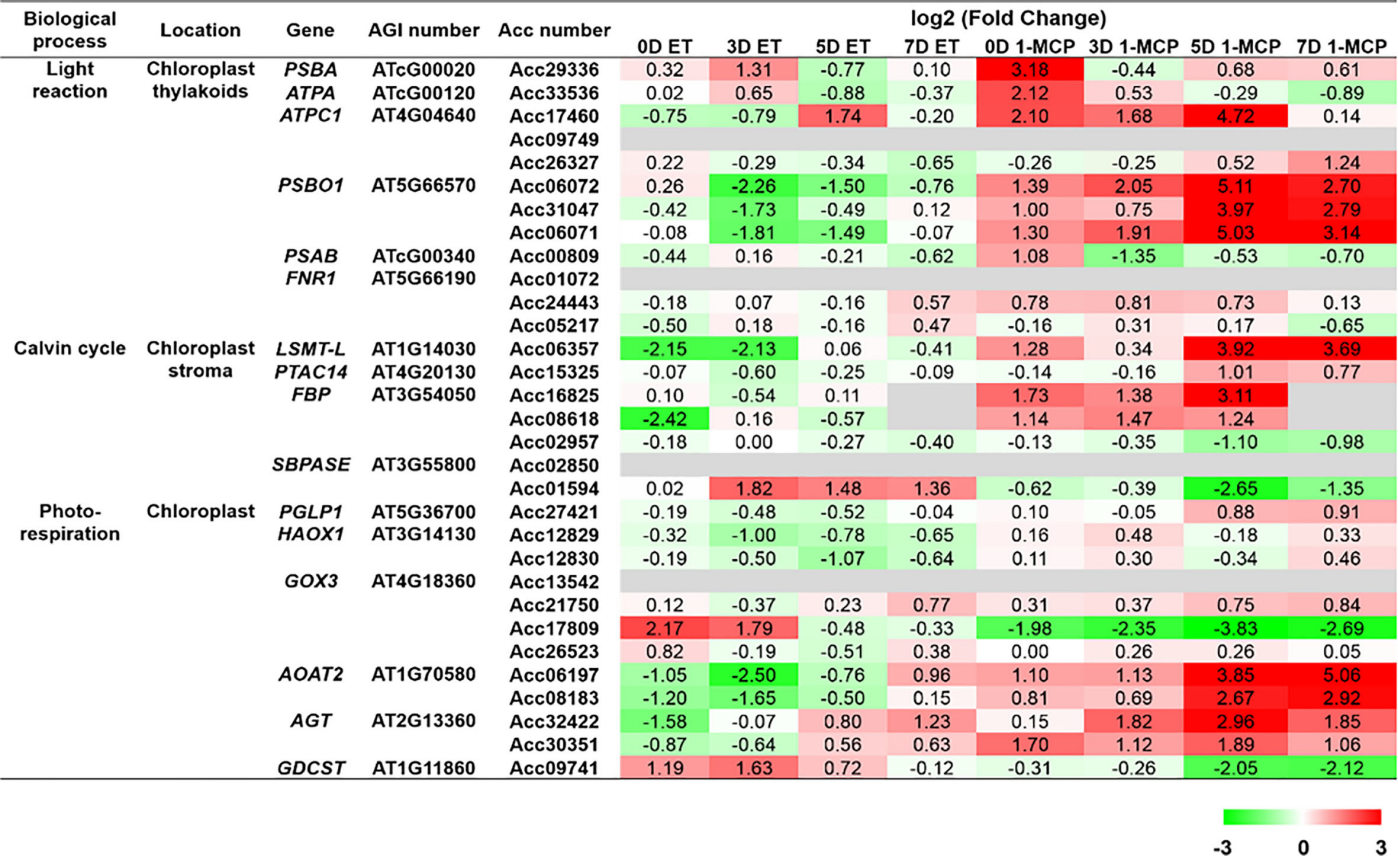
Normalized heatmap showing expression levels of photosynthesis-related genes upon ET and 1-MCP treatment along ripening time course. Thirty-one genes related to photosynthesis processes, like light reaction, Calvin cycle, and photorespiration, were found from the *Actinidia chinensis* genome database, and their expression levels were compared between ET-treated samples and 1-MCP-treated samples along ripening time course (0 days [0D], 3 days [3D], 5 days [5D], and 7 days [7D]). Twenty-seven of 31 total genes (87%) except four genes (Acc01594, Acc17809, Acc26523, and Acc09741) were highly upregulated upon 1-MCP treatment in at least one time point during ripening compared to those of ET-treated samples. ET, ethylene; 1-MCP, 1-methylcyclopropene; gene, *Arabidopsis* gene name; AGI, *Arabidopsis* gene index; Acc, *A. chinensis*.

To grasp the biological categories affected by ET or 1-MCP treatment at individual time points, the GO analysis of the aforementioned DEGs was performed at individual time points (**Figures 4A–D**). First, DEGs downregulated by ET and inversely upregulated by 1-MCP were analyzed to identify the top 10 enriched GO terms at the 0D, 3D, 5D, and 7D time points, respectively. As a result, two biological processes related to photosynthesis and gene silencing were significantly enriched in the top 10 GO terms (**Figures 4A–D**). The result that the GO term related to photosynthesis was highly enriched in 1-MCP-treated samples at all time points reflected that one role of 1-MCP is to robustly block chlorophyll degradation and/or sustain photosynthetic light reactions and photosynthesis activity during fruit storage. Therefore, we collected the photosynthetic light reaction/photosynthesis-related genes from the KGD to examine their transcriptional patterns. A BLAST search using sequence information of photosynthesis-related genes obtained from the model plant *Arabidopsis* identified 31 genes (**Supplementary Table S5**). As shown in **Figure 5**, most of the photosynthesis-related genes (27 out of 31) except four genes (Acc01594, Acc17809, Acc26523, and Acc09741) were highly upregulated upon 1-MCP treatment in at least one time point during ripening compared to those of ET-treated samples. It indicated that the photosynthetic light reaction/photosynthesis process is one of the main targets of 1-MCP in kiwifruit.

### 3.7 GO categories phenylpropanoids and amino acid biosynthesis are highly affected in ET-treated kiwifruit samples

Next, we also performed GO enrichment analysis with the list of DEGs showing upregulation by ET and downregulation by 1-MCP from 0D to 7D (**Figures 4A–D**). Phenylpropanoids (also referred to as cinnamic acid) biosynthesis and amino acid biosynthesis (cysteine and phenylalanine), both GO terms related to secondary metabolisms, were substantially detected in the top 10 GO terms in the samples at all time points (**Figures 4A–D**). Phenylpropanoids are a group of secondary metabolites with diverse roles in plant development (i.e., cell wall formation) as well as fruit ripening (i.e., pigmentation) (Singh et al., 2010). Furthermore, they also play an important role in stress responses, such as the increased production of antioxidants in plants to counteract ultraviolet radiation-induced oxidative stress.

### 3.8 Expression of phenylpropanoid pathway genes was substantially affected by ET and 1-MCP treatment during ripening

**Figure 6:**
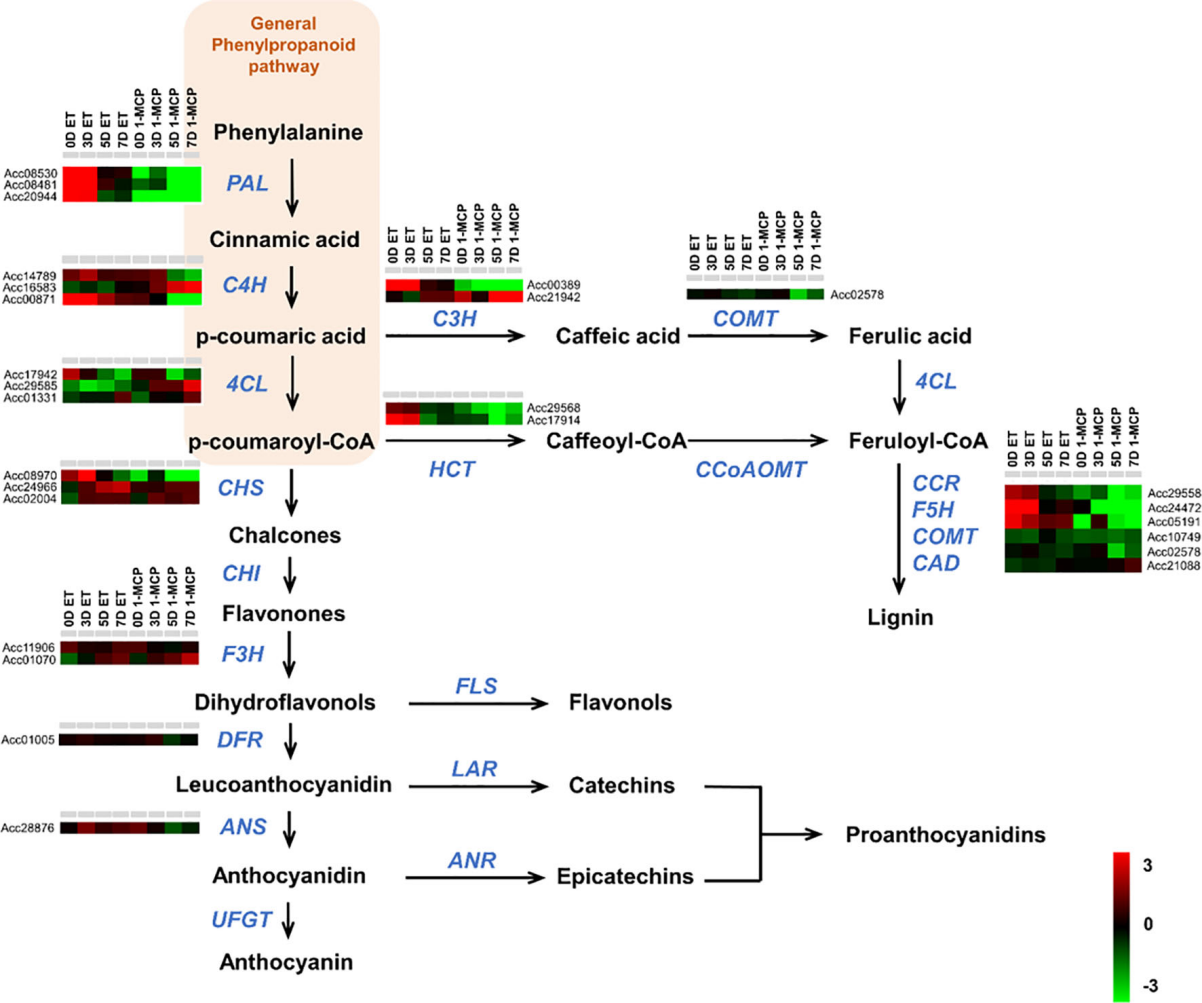
Expression profiles of phenylpropanoid pathway genes upon ET and 1-MCP treatment along ripening time course. Schematic diagram showing phenylpropanoid pathways beginning from general phenylpropanoid pathway constituting *PAL*, *C4H*, and *4CL* genes and then subsequently branched into different biosynthesis pathways for anthocyanins, proanthocyanidins, and lignin. Gene names responsible for each step of the phenylpropanoid pathway were indicated with italicized blue-colored letters. Expression levels of genes related to each step of the phenylpropanoid biosynthesis pathway were presented as a heatmap. Red cube represents upregulated gene expression, and green cube represents downregulated gene expression compared to the level of the control sample. ET, ethylene; 1-MCP, 1-methylcyclopropene.

Phenylpropanoids have a relatively simple structure and are synthesized by the shikimic acid pathway and the cinnamic acid pathway through sequential catalytic reactions starting from the condensation of 2-phosphoenolpyruvate and D-erythrose 4-phosphate. To check the expression pattern of the phenylpropanoid metabolic pathway genes, we first collected the phenylpropanoid metabolic pathway genes from the KGD. A total of 27 genes were identified as genes related to the phenylpropanoid pathway from the *Actinidia chinensis* genome (**Supplementary Table S6**). Many genes were upregulated in ET-treated samples compared to those in 1-MCP-treated samples over the entire time course. In particular, genes involved in the lignin biosynthesis pathway, an important branch of the general phenylpropanoids pathway, were significantly upregulated by the ET treatment in comparison to the 1-MCP treatment (**Figure 6**). For example, three *PAL* homologs, three *C4H* homologs, two *HCT* homologs, one *COMT* homolog, and three *CCR* homologs exhibited markedly higher expression in ET-treated samples than in 1-MCP-treated samples (**Figure 6**). To validate the RNA-seq result, we performed qRT-PCR analysis on a phenylalanine ammonia-lyase (named *AcPAL1)* in the phenylpropanoid pathway of *A. chinensis* (**Supplementary S5B**). Expression of *AcPAL1* (Acc08530) was substantially upregulated in ET-treated samples at all time points compared to levels in the control (GC). Meanwhile, 1-MCP-treated samples showed low expression of *AcPAL1* at all tested time points. Detailed information on the RNA-seq read count of each gene is listed in **Supplementary Table S6**. Taken together, these results indicate that phenylpropanoid pathway genes, particularly lignin biosynthesis pathway genes, are one target of the ET hormone during the ripening of kiwifruit.

### 3.9 Cysteine amino acid biosynthesis pathway was affected by ET and 1-MCP treatment during kiwifruit ripening

Another GO term was related to amino acid biosynthesis (phenylalanine and cysteine). As mentioned above, phenylalanine is an amino acid derived from the shikimate pathway (Tohge et al., 2013) and is used as a precursor for the phenylpropanoid biosynthesis pathway, which synthesizes diverse metabolites like flavonols, dihydrochalcones, flavones, flavanones, and anthocyanins (Treutter, 2006). Another amino acid, cysteine, is one of the precursors for the synthesis of various sulfur-containing volatiles produced during fruit ripening (Panpetch and Sirikantaramas, 2021). For example, in ripening fruit, cysteine is used to generate defense-related compounds, such as glutathione, glucosinolates, camalexin, and ET hormone (Romero et al., 2014). The GO term related to cysteine biosynthesis was enriched at the later storage times (5D and 7D), whereas the GO term associated with phenylpropanoid (cinnamic acids) biosynthesis was observed at the relatively earlier time points (0D and 3D).

### 3.10 Transcriptional dynamics of cysteine biosynthetic pathway genes between ET- and 1-MCP-treated samples

**Figure 7:**
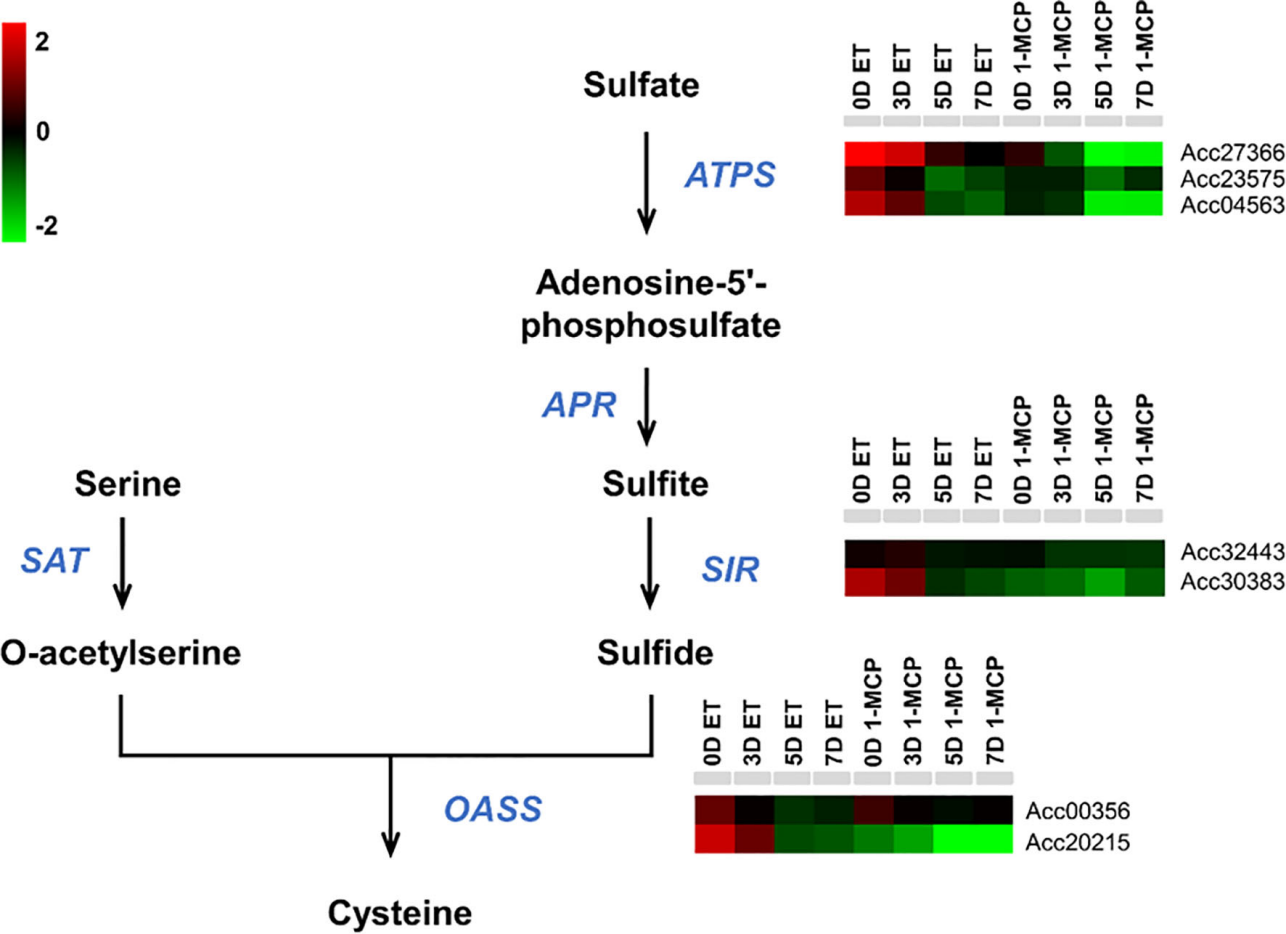
Expression profiles of cysteine biosynthesis pathway genes upon ET and 1-MCP treatment along ripening time course. Schematic diagram showing cysteine biosynthesis pathway from a precursor, sulfate. Gene names responsible for each step of the cysteine biosynthesis pathway were indicated with italicized blue-colored letters. Expression levels of genes related to each step of the cysteine biosynthesis pathway were presented as a heatmap. Red cube represents upregulated gene expression, and green cube represents downregulated gene expression compared to the level of the control sample. ET, ethylene; 1-MCP, 1-methylcyclopropene.

Cysteine is a reduced donor compound involved in the biosynthesis of many essential secondary metabolites (Romero et al., 2014). Having established that the cysteine biosynthesis pathway was also a significantly enriched top 10 GO term using the list of DEGs (upregulated by ET and downregulated by 1-MCP), particularly at relatively late time points (5D and 7D) (**Figures 4C, D**), we examined the transcriptional profiles of genes related to cysteine biosynthesis in kiwifruit during ripening. Among a total of seven genes related to cysteine biosynthesis, six genes except Acc00356 were commonly upregulated by ET and downregulated by 1-MCP treatment during ripening (**Figure 7**; **Supplementary Table S7**). The RNA-seq data were validated by the qRT-PCR analysis performed on a cysteine biosynthesis pathway gene, *AcCAS-C1* (Acc20215) (**Supplementary Figure S5C**). It indicates that kiwifruit ripening is accompanied by cysteine biosynthesis.

### 3.11 Ripening of kiwifruit is accompanied by induction of ET biosynthetic genes and ET signaling

**Figure 8:**
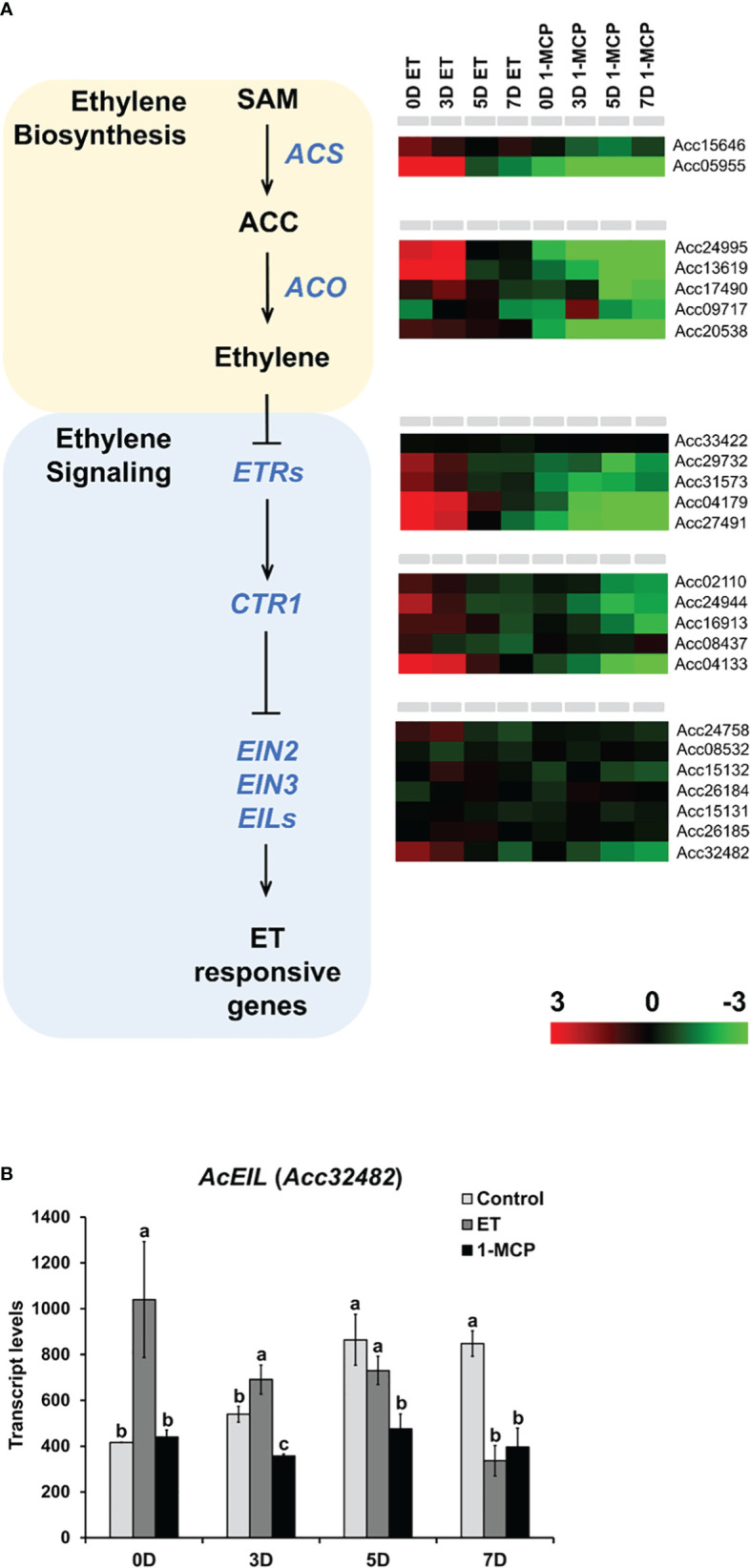
Expression profiles of ET signaling and biosynthesis pathway genes upon ET and 1-MCP treatment along ripening time course. **(A)** Schematic diagram showing ET biosynthesis (pink box) and ET signaling (blue box) beginning from a precursor, S-adenosyl methionine (SAM). Gene names responsible for each step of ET biosynthesis and signaling pathway were indicated with italicized blue-colored letters. Expression levels of genes related to each step of ET biosynthesis and signaling pathway were presented as a heatmap. Red cube represents upregulated gene expression, and green cube represents downregulated gene expression compared to the level of the control sample. ET, ethylene; 1-MCP, 1-methylcyclopropene. **(B)** Transcript levels of *AcEIL* (Acc32482) in the control, ET-treated, and 1-MCP-treated samples along four different time course (0D, 3D, 5D, and 7D). ET, ethylene; 1-MCP, 1-methylcyclopropene. Data represent the mean of the transcript values from three biological replicates with error bars showing standard deviation (n=3). One-way ANOVA and *post-hoc* Tukey’s test (p < 0.05) was applied to calculate the statistical significance, and significant difference was indicated in the figures by different letters (p < 0.05).

ET plays a pivotal role in the ripening of many fruits. In our study, treatment with ET and its inhibitor 1-MCP resulted in dynamic transcriptional changes in kiwifruit during ripening. Thus, we analyzed the expression profiles of the ET biosynthetic pathway genes as well as ET signaling genes in ET-treated and 1-MCP-treated samples over the ripening course. In total, 6 and 17 genes related to ET biosynthesis and ET signaling, respectively, were identified from the *A. chinensis* genome (**Figure 8**; **Supplementary Table S8**). Two *ACS* and five *ACO* homolog genes displayed markedly higher expression in ET-treated samples than in 1-MCP-treated samples over the ripening course. Among the ET signaling genes, five ET receptors and five CTR1 homologs commonly showed upregulated expression in ET-treated samples, particularly at early time points (0D and 3D), compared to the 1-MCP-treated samples (**Figure 8A**). Among the ET signaling transcription factor genes, such as *EIN2*, *EIN3*, and *EIN3-like (EIL)* homologs, five genes (Acc24758, Acc15132, Acc15131, Acc26185, and Acc32482) out of a total of seven genes showed differential expression in ET-treated and 1-MCP-treated samples, but the other two genes (Acc08532 and Acc26184) did not show a dramatic difference between the ET- and 1-MCP-treated samples (**Figure 8A**; **Supplementary Table S8**). To validate the RNA-seq result, we performed qRT-PCR analysis on an ET signaling gene (named *AcETR2*, Acc04179) and an ET biosynthesis gene (named *AcACS6*, Acc05955) (**Supplementary Figure S5D**). A similar pattern to the RNA-seq data was shown in the qRT-PCR result. Given the fact that EIN3 and EILs play an important role as transcription factors in ET-mediated signaling, *AcEIN3/AcEIL* homologs might play a crucial role as transcription factors in the ET-mediated ripening in kiwifruit. Among *AcEIN3/AcEIL* signaling transcription factors, most drastic expressional difference between ET- and 1-MCP-trreated samples was observed in the *AcEIL* (Acc32482), highly upregulated by ET and downregulated by 1-MCP treatment in the early storage time points (0D~3D) (**Figure 8B**). It suggested that *AcEIL* homolog (Acc32482) might play an important role in the ET signaling cascade in the early ripening stage of kiwifruit. Taken together, most ET signaling and ET biosynthesis pathway genes are positively regulated by ET treatment but negatively affected by 1-MCP treatment during ripening.

## 4 Discussion

To elucidate the molecular details underlying ET-mediated ripening of kiwifruit, we analyzed transcriptomic changes by the treatment of ethylene (ET) or 1-MCP, an artificial ethylene inhibitor along the different time course of storage. We found that ET and 1-MCP targets on metabolic pathway genes related to photosynthesis, the cell wall formation, phenylpropanoids, sugar and cysteine biosynthesis Furthermore, ET biosynthetic and signaling pathway genes oppositely affected by ET and 1-MCP treatment were identified in the course of kiwifruit ripening.

In the measurement of endogenous ET production in control, ET-treated, and 1-MCP treated kiwifruit samples, we unexpectedly observed that maximum peak level of ET in the ET-treated samples was similar to that of the control (**Figure 1C**). Regarding this observation, it is worthy to note that ET-treated samples exhibited the earlier ET production than the control sample and the softening rate was accelerated. It can be inferred that the ET production peak in the ET-treated samples might be around the 6th day, not the 7th day. Because measurement of ET was conducted every 2 days, it is likely that ET production peak time might be skipped. This data indicate that exogenous ET treatment might have a positive impact on kiwifruit ripening within 7 days in our tested condition. Meanwhile, 1-MCP treatment displayed constantly decreased ET production throughout all tested ripening period (BT~14 days), confirming that 1-MCP play a negative impact on the ET biosynthesis in kiwifruit ripening stage.

A recent paper performed transcriptomic analysis to dissect effects of ET and 1-MCP on kiwifruit fruit ripening (Salazar et al., 2021). This paper showed that ET greatly influences sugar metabolism and photosynthesis biosynthetic pathways which was also observed in our study. However, this research differs from our study in terms of sample time points. That is, RNA-seq analysis of the previous study was performed only at two time points, 4 days and 13 days after treatment, whereas our transcriptome analysis was performed at four different time points (0, 3, 5, and 7 days after treatment). Through this delicate analysis, we tried to capture the comprehensive transcriptomic profiles spanning early stage of ripening (0 and 3 days) to later ripening (5 and 7 days). It was expected that our transcriptomic data might capture more molecular details than the previous paper, particularly on the dynamic transcriptional events occurring during early stage of ripening. As a result, GO analysis using DEGs found in this study identified the target genes by ET and 1-MCP, which are related to diverse biological pathways such as photosynthesis, pigment change (e.g. chlorophyll), sugar metabolism, cell wall metabolism, cysteine biosynthesis, and phenylpropanoids (i.e. lignin) compared to the previous paper.

Interestingly, the GO term ‘photosynthesis’ was consistently detected at all (0D−7D) time points in our GO analysis. This result corroborates the previous reports that ET accelerates chlorophyll degradation, thus resulting in changes in fruit color (Iqbal et al., 2017; Salazar et al., 2021; Kapoor et al., 2022). Thus, it is likely that photosynthesis biosynthetic pathway might be the one of major target by ET-mediated ripening process. In a consistency, previous studies reported that ET signaling factors like APETALA2/ethylene responsive factor (AP2/ERF) family genes play an important role in the fruit ripening through regulation of genes related to photosynthesis, chlorophyll metabolism during fruit ripening (Zhai et al., 2022).

Another GO term related to gene silencing by RNA was also significantly found at the early time points (0D and 3D), suggesting that post-transcriptional gene silencing by non-coding RNAs (ncRNAs) like miRNAs might play an important role in the ET-mediated ripening process in kiwifruit. Involvement of ncRNAs-mediated gene silencing has been reported in many flesh fruit so far (Wang et al., 2022b). For example, a genetic study using cultivated and wild tomato species revealed that small interfering RNAs (siRNAs)-mediated gene silencing and DNA methylation contribute to heritable transgressive phenotypes (Shivaprasad et al., 2012). In case of kiwifruit, a high-throughput small RNA-seq analysis unveiled that differential expression and repression of *MYB* genes which are targeted by small RNAs like miR828 and its phased small RNA AcTAS4-D4(-) is responsible for variable accumulation of anthocyanin in kiwifruit (Wang et al., 2022a). In the last decade, advance of high-throughput sequencing technology facilitated genome-wide profiling of small RNAs in ripening fruits including tomato, grape, banana, and olive and suggested that small RNA-mediated gene silencing and DNA methylation/demethylation might contribute to the developmental transition in flesh fruits (Sato et al., 2012; Belli Kullan et al., 2015; Bi et al., 2015; Carbone et al., 2019; Wang et al., 2022b). Recent studies strongly suggest that ncRNAs play an important role in the regulation of fruit ripening process. Therefore, elucidation of ncRNAs involved in the kiwifruit ripening might broaden our understanding on the molecular mechanism underlying kiwifruit ripening.

Considering that exogenous ET treatment exhibited a positive effect on the expression of ET signaling and ET biosynthesis pathway genes, it is likely that exogenous ET treatment might further reinforce the biosynthesis of endogenous ET in ripening fruit. However, in our study, endogenous ET production was temporarily enhanced at an early time point (3D) in ET-treated samples but did not display a significant increase in later stages (5D and 7D) in ET-treated samples (**Figure 1C**). Hence, further clarification is needed to determine whether kiwifruit has a feedback regulation of ET production upon the treatment of exogenous ET. One plausible explanation would be that exogenous ET treatment triggered transcriptional activation of ET signaling and ET biosynthesis genes; negative feedback regulation might act at the translational or post-translational level to maintain homeostasis of endogenous ET in kiwifruit. Regarding this hypothesis, a study using the model plant *Arabidopsis* reported that ET signaling and biosynthesis are finely modulated at the protein level by EIN3-binding F-box proteins (EBF1 and EBF2), which interact with EIN3 and EIL1 and destabilize EIN3/EIL1 *via* ubiquitination-mediated protein degradation, thus providing negative feedback regulation (An et al., 2010). In addition to studying whether kiwifruit has EBF1/2-mediated feedback regulation in endogenous ET biosynthesis, it would also be worth studying whether different genotypes and storage conditions (i.e., temperature) influence the effect of exogenous ET and 1-MCP treatments on kiwifruit ripening.

Previous studies reported that some kiwifruit ET biosynthetic genes such as *AC-SAM1*, *AC-SAM2*, *AC-ACO1*, and *AC-ACO2* and ET signaling factor genes like *KWACO1* and *KWACS1* genes were activated and required for the ET biosynthesis (Ilina et al., 2010; Mworia et al., 2010). In addition, some ET signaling factors including AdEIN3-like (AdEILs) and ethylene response factors (ERFs) were reported to be dynamically regulated in ripening of kiwifruit (*Actinidia deliciosa*) (Yin et al., 2010). In our study, we also observed that 5 ET biosynthetic genes (2 *AcACS* and 3 *AcACO* homologs) and 7 ET signaling factor genes (4 *AcETRs*, 2 *AcCTRs*, and 1 *AcEIL* homolog) were up-regulated in ET-treated samples than control samples (**Figure 8A**). In contrast, 7 ET biosynthetic genes (2 *AcACS* and 5 *AcACO* homologs) and 9 ET signaling factor genes (4 *AcETRs*, 4 *AcCTRs*, and 1 *AcEIN3* homologs) were down-regulated in 1-MCP-treated samples along the ripening time course. It indicates that expression of both ET biosynthetic as well as ET signaling factors are dynamically modulated by ET and 1-MCP treatment during ripening. Particularly, this study found that *AcEIL* (Acc32482) was highly up-regulated by ET and down-regulated by 1-MCP at early ripening stage (0D~3D), suggesting that it might play an important role in ET-mediated ripening process of kiwifruit (**Figure 8B**). CRISPR-Cas9 Genome editing on key ET signaling factor genes like *AcEIN3/AcEIL* homologs (e.g. *AcEIL*) in kiwifruit might be helpful to unveil the molecular mechanism underlying ET-mediated ripening process in kiwifruit. Collectively, our transcriptomic study unveiled the molecular targets by ET and its antagonist, 1-MCP, such as ET-targeted metabolisms and ET-targeted metabolic genes in kiwifruit during ripening and might be valuable for further investigation on kiwifruit fruit ripening.

## Data availability statement

The datasets presented in this study can be found in online repositories. The names of the repository/repositories and accession number(s) can be found below: https://www.ncbi.nlm.nih.gov/genbank/, GSE214748.

## Author contributions

JL and D-HK designed the study. DC and JC prepared all materials. JC and K-JP analyzed firmness, ethylene, and sugar contents. DC and CK performed molecular experiments. DC and D-HK performed the transcriptome analysis. JL and D-HK supervised the experiments and wrote the manuscript. All authors contributed to the manuscript and approved the final version of the manuscript to be published.
