# Supplementary material for: Transcriptomic analysis of effects of 1-methylcyclopropene (1-MCP) and ethylene treatment on kiwifruit (Actinidia chinensis) ripening
Source: Front Plant Sci. 2023 Jan 5;13:1084997. doi: 10.3389/fpls.2022.1084997 (PMC9849763; doi:10.3389/fpls.2022.1084997)
Supplement: Supplementary file 2 [file DataSheet_2.docx]

Supplementary Material

# Supplementary Figures


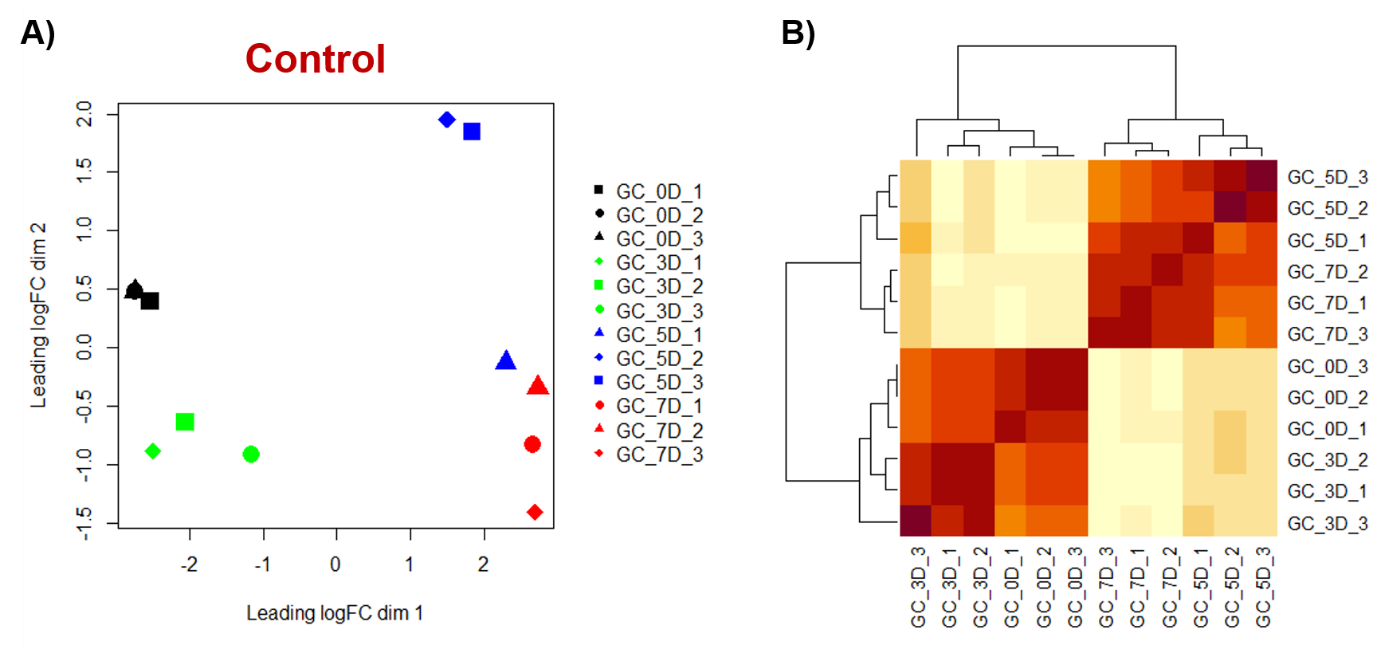


**Supplementary Figure S1. Quality check of RNA-seq reads of control kiwifruit samples during the ripening course.** (**A**) Multi-dimensional scaling (MDS) analysis of RNA-seq reads using green kiwifruit control (GC) along ripening time course, 0D (black symbols), 3D (green symbols), 5D (blue symbols), and 7D (red symbols). MDS plot showed samples within the same group had similar gene expression profiles and were significantly different between groups. (**B**) Heatmap of the Pearson correlation matrix between each sample for pairwise sample comparison. In the control green kiwifruit (GC), 0D and 3D time point samples exhibited a clustering. Meanwhile, 5D and 7D time point samples also showed similar clustering in profiles of RNA-seq reads. The color key was adjusted based on the log_2_-centered values to show differences. Dendrograms show distances between repeat samples. 0D, 0 days; 3D, 3 days; 5D, 5 days; 7D, 7 days.


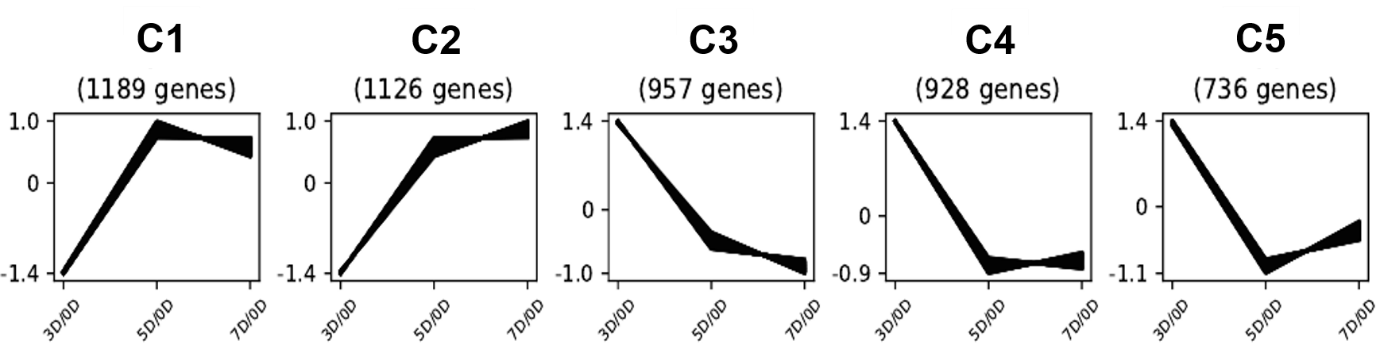


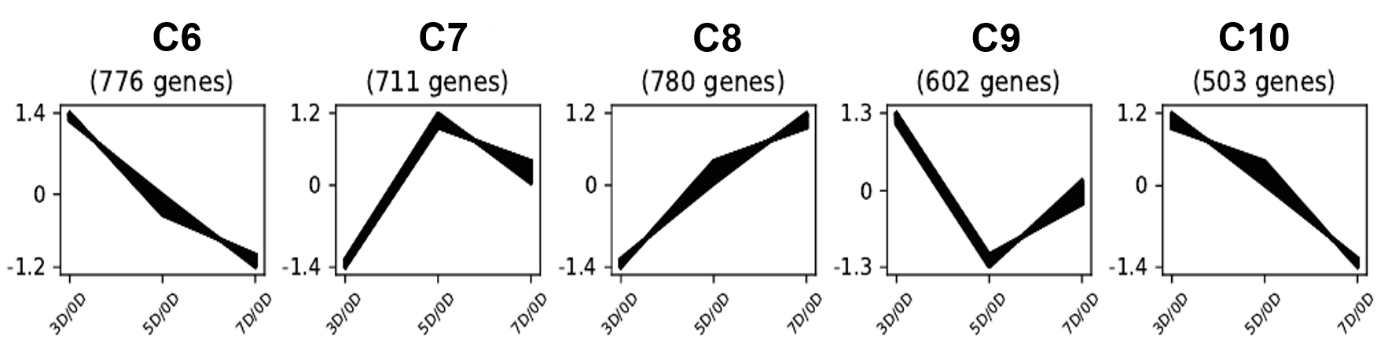


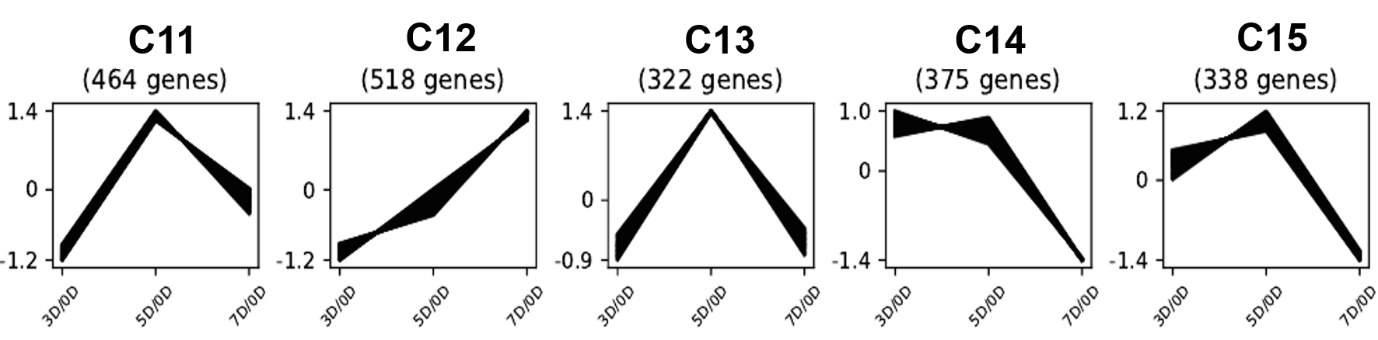


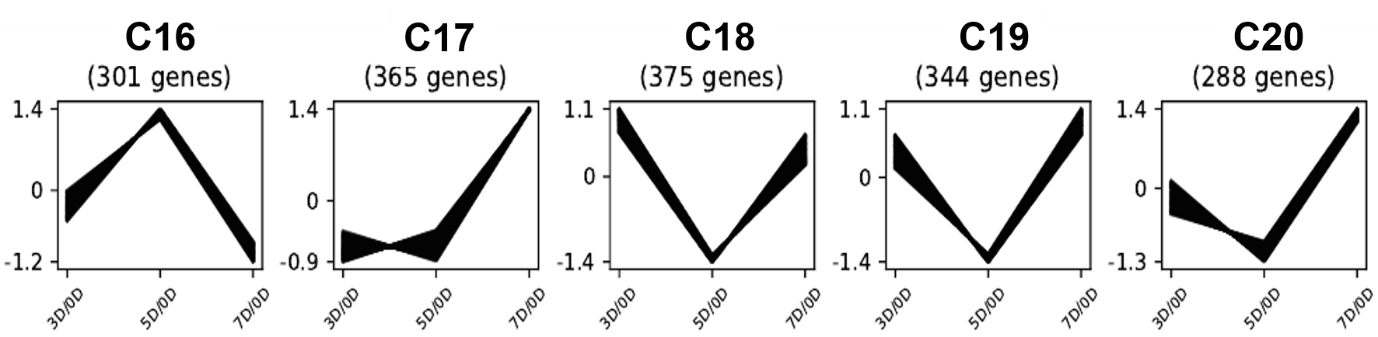


**Supplementary Figure S2. Hierarchical clustering of DEGs along ripening course based on the expression patterns.** A total of 20 clusters were generated based on transcriptional patterns of DEGs during the ripening time course. 3D/0D, expression level of treated sample after storage for 3 days (3D) compared to 0 days (0D); 5D/0D, expression level of treated sample after storage for 5 days (5D) compared to 0 days (0D); 7D/0D, expression level of treated sample stored for 7 days (7D) compared to 0 days (0D). Number of genes belonging to each cluster was shown with parenthesis below individual cluster number. DEG, differentially expressed gene.


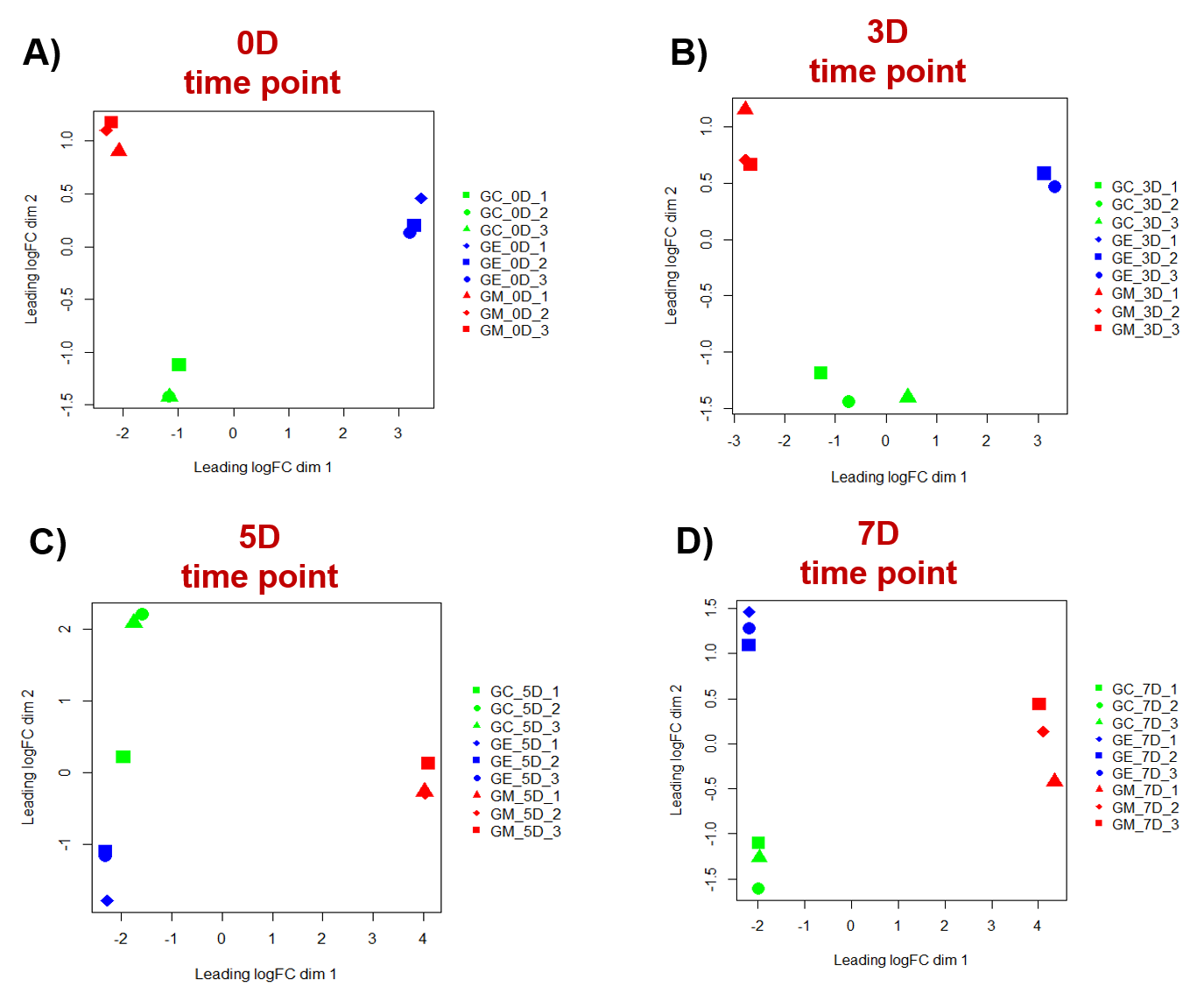


**Supplementary Figure S3.** Multi-dimensional scaling (MDS) analysis of RNA-seq reads using RNA-seq reads of time-course green control kiwifruit (GC, green color symbols), ET-treated green kiwifruit (GE, blue color symbols), and 1-MCP-treated green kiwifruit (GM, red color symbols) stored for (**A**) 0 days (0D), (**B**) 3 days (3D), (**C**) 5 days (5D), and (**D**) 7 days (7D). These MDS plots indicated that samples within the same group had similar gene expression profiles and were significantly different between groups.


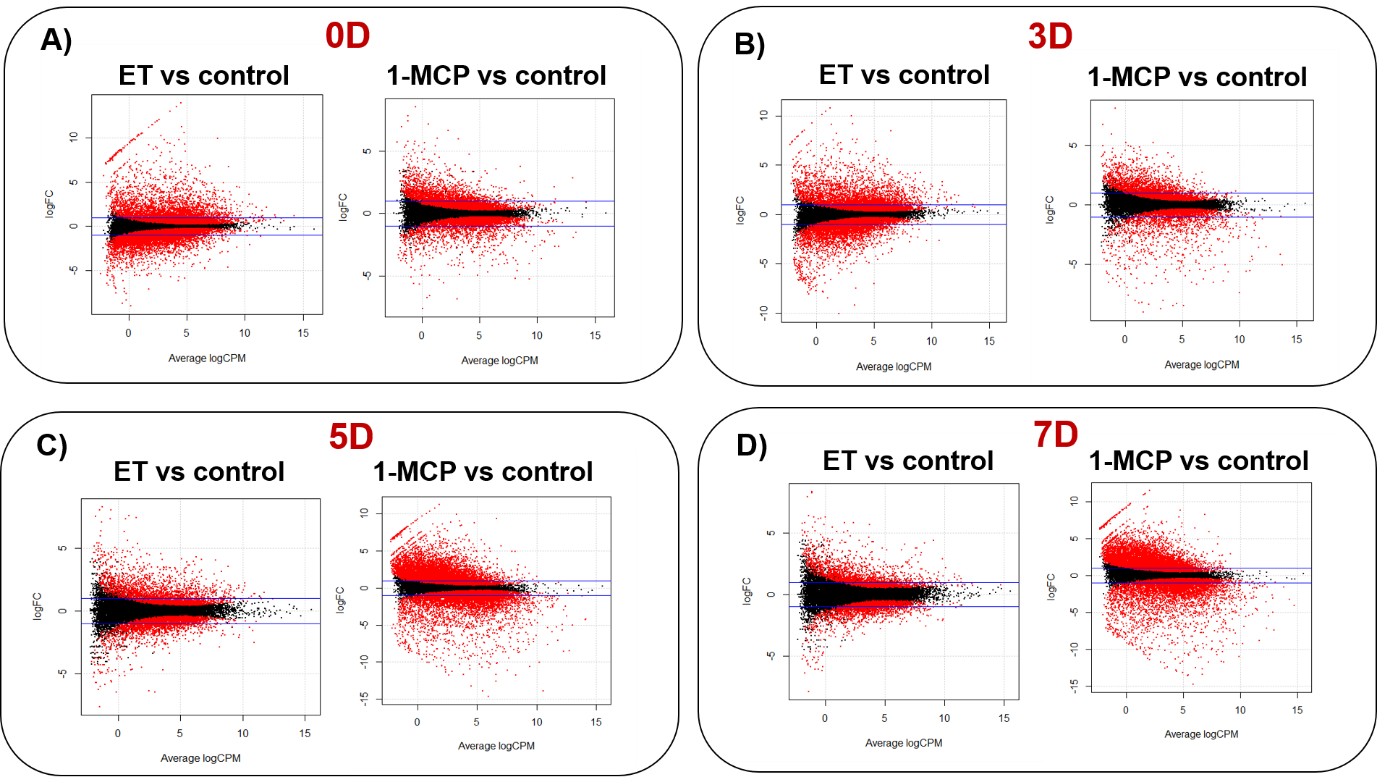


**Supplementary Figure S4.** Mean average (MA) plots of DEGs between control and ET-treated samples or between control and 1-MCP-treated samples at (**A**) 0 days (0D), (**B**) 3 days (3D), (**C**) 5 days (5D), and (**D**) 7 days (7D). The *x*-axis represents average expression, logCPM (log_2_-transformed expression value), and the *y*-axis represents the value of log_2_-transformed fold change. Red dots indicate DEGs between control and ET-treated samples or between control and 1-MCP-treated samples. Black dots indicate non-DEGs. DEG, differentially expressed gene; ET, ethylene; 1-MCP, 1-methylcyclopropene.


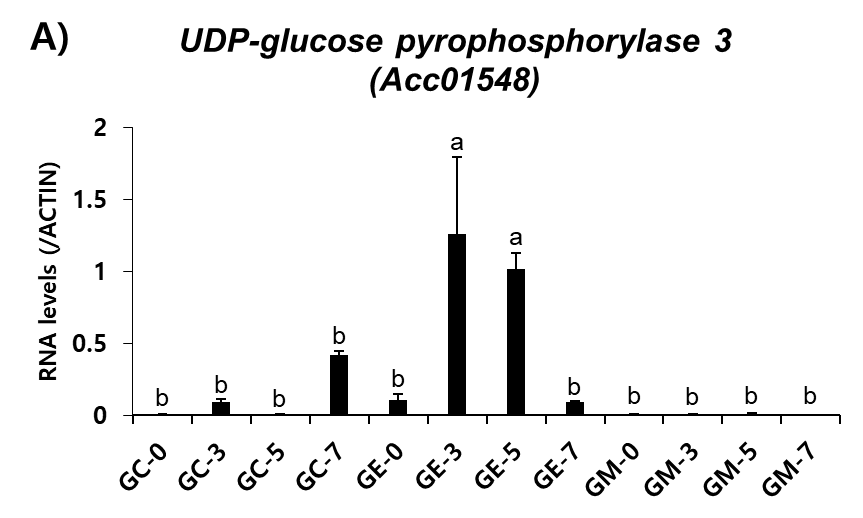

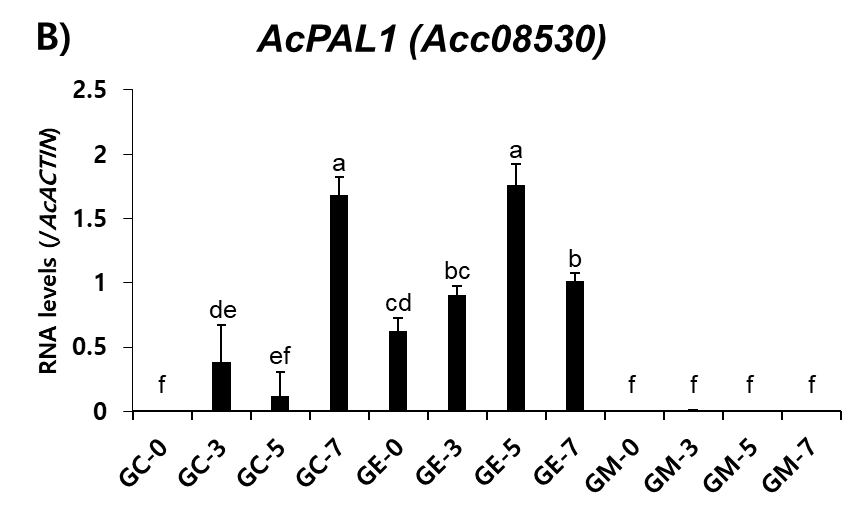


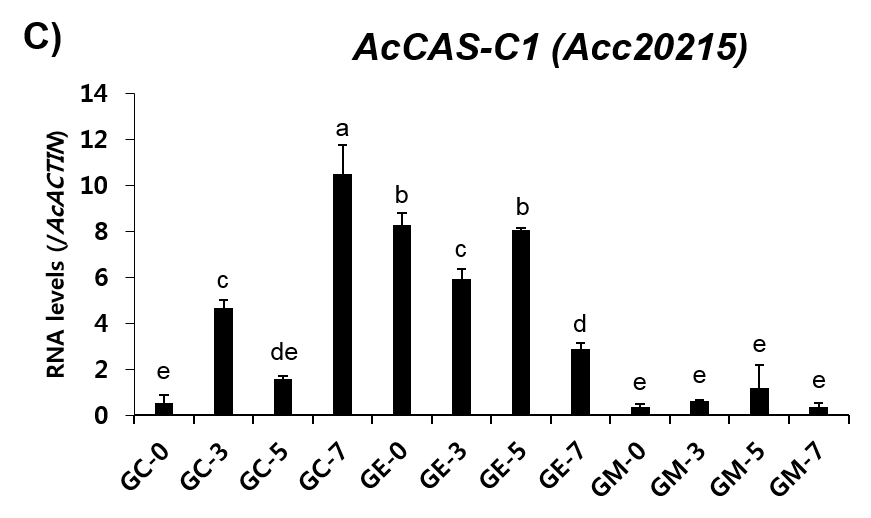


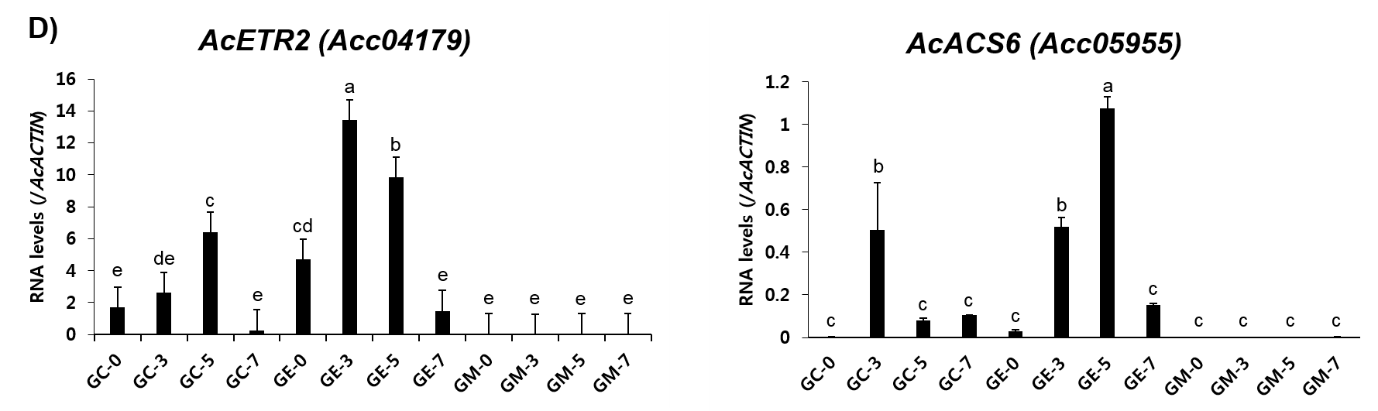


**Supplementary Figure S5.** Result of qRT-PCR analysis on genes related to the (**A**) cell wall formation, (**B**) phenylpropanoids biosynthesis, (**C**) cysteine biosynthesis, and (**D**) ET signaling (left) and ET biosynthesis pathway (right). **A−D** Data were presented as mean ± standard deviation (*n* = 3). Significant difference was determined by one-way ANOVA and Tukey’s post hoc test (*p* < 0.05). ET, ethylene.

# Supplementary Tables

**Supplementary Table S1.** Primer sequences used in this study

**Supplementary Table S2.** List of genes belonging to individual clustered groups analyzed by hierarchical clustering analysis

**Supplementary Table S3.** Information on differentially expressed genes (DEGs) affected by ET and 1-MCP treatment at each time point (0, 3, 5, and 7 days; 0D, 3D, 5D, and 7D). ET, ethylene; 1-MCP, 1-methylcyclopropene

**Supplementary Table S4.** Expression levels of 131 genes related to cell wall formation in *Actinidia chinensis* genome

**Supplementary Table S5.** Expression levels of 31 photosynthetic light reaction/photosynthesis-related genes in *Actinidia chinensis* genome

**Supplementary Table S6.** Expression levels of 27 genes related to phenylpropanoids in *Actinidia chinensis* genome

**Supplementary Table S7.** Expression levels of seven genes related to cysteine biosynthesis in *Actinidia chinensis* genome

**Supplementary Table S8.** Expression level of six ET biosynthetic genes and 17 ET signaling genes identified in *Actinidia chinensis* genome. ET, ethylene.
